# Ultimate opportunists—The emergent *Enterocytozoon* group Microsporidia

**DOI:** 10.1371/journal.ppat.1007668

**Published:** 2019-05-02

**Authors:** Grant D. Stentiford, David Bass, Bryony A. P. Williams

**Affiliations:** 1 International Centre of Excellence for Aquatic Animal Health, Centre for Environment Fisheries and Aquaculture Science, Weymouth Laboratory, Weymouth, Dorset, United Kingdom; 2 Centre for Sustainable Aquaculture Futures, College of Life and Environmental Sciences, University of Exeter, Exeter, United Kingdom; 3 Department of Life Sciences, The Natural History Museum, London, United Kingdom; 4 Biosciences, College of Life and Environmental Sciences, University of Exeter, Exeter, United Kingdom; Geisel School of Medicine at Dartmouth, UNITED STATES

## Opportunistic invaders in all biomes

The Microsporidia are a diverse intracellular parasite phylum infecting everything from single-celled protists to humans in all major global biomes. Its 200 or more known genera are grouped into at least five major clades, with vast numbers of taxa in currently unknown hosts remaining undiscovered [1]. One clade that contains perhaps the most intriguing genera within the phylum, the *Enterocytozoon* group Microsporidia (EGM), includes parasites that infect the cells (and sometimes the nuclei) of invertebrates and fish hosts from aquatic habitats, terrestrial birds and mammals, and human patients with underlying immune-suppressive conditions such as HIV/AIDS [1]. The EGM was not known prior to the HIV/AIDS pandemic in the 1980s but now contains the most prevalent human microsporidian pathogen, *Enterocytozoon bieneusi* [2]. It is also associated with recent emergent diseases in domestic and companion animals [3] and outbreaks in intensive rearing operations for marine shrimp [4], fish [5], and other wildlife [6]. Certain members of the group exhibit life cycles that passage between invertebrate and vertebrate hosts [7]. It has been proposed that emergence of disease caused by members of this group over the past 5 decades may be indicative of inherent stressors acting upon those biomes in which their hosts exist. Under these suboptimal conditions, opportunistic infections with enhanced potential to cross host taxonomic boundaries have led to increased prevalence and host range in the EGM [1].

## An enigmatic clade

**Fig 1:**
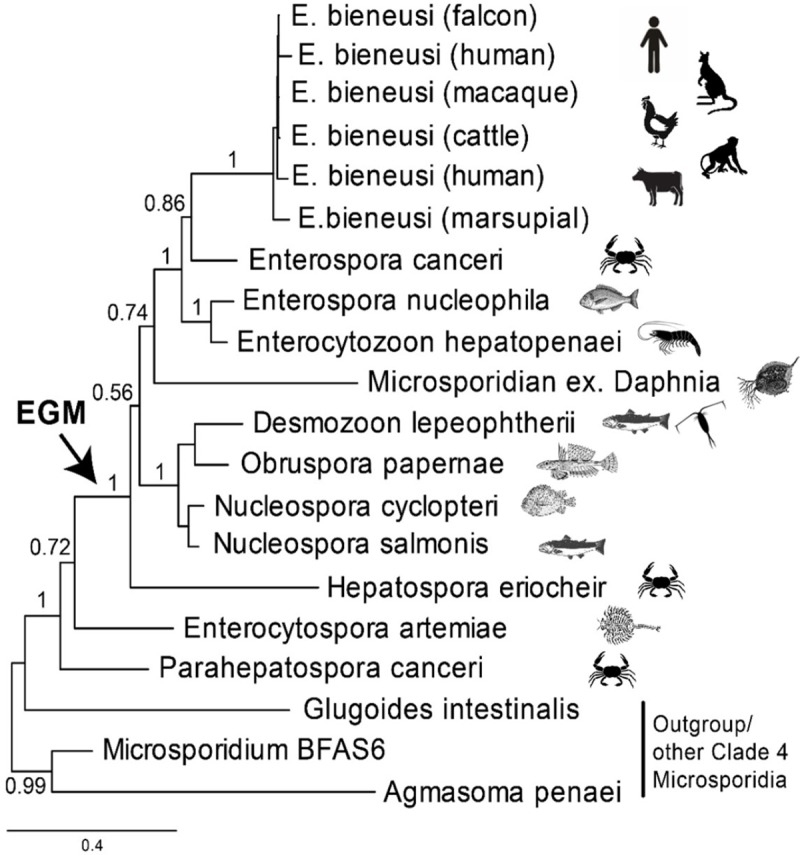
Phylogeny and host range of known members of the EGM. Except for *E*. *bieneusi* infecting humans, terrestrial mammals, and birds, all other members of the EGM infect aquatic hosts. Erection of the genus *Enterocytozoon* coincided with emergence of its type species *E*. *bieneusi* during the HIV/AIDS pandemic in the 1980s. Subsequent description of proposed cogeneric EGM genera (*Nucleospora*, *Paranucleospora/Desmozoon*, *Enterospora*, *Hepatospora*, *Obruspora*) and closely related genera (e.g., *Enterocytospora* and *Parahepatospora*) have occurred over the past 3 decades. In most cases, emergence has been reported in domesticated terrestrial and aquatic food animals. However, certain EGM and related taxa have also been described infecting wild-animal hosts (e.g., foxes, primates, crabs), some of which reside in invasive populations outside of their native ranges. In at least one case (*Paranucleospora/Desmozoon*), the parasite is known to cycle between an invertebrate (copepod crustacean) and fish (salmon) host, raising the prospect of the capacity for trophic transfer in other members of the EGM. The Bayesian phylogenetic analysis was conducted using MrBayes version 3.2.5 [27]. The evolutionary model applied included a GTR substitution matrix, a four-category autocorrelated gamma correction, and the covarion model for 4 million generations, 1 million of which were discarded as burn-in. EGM, *Enterocytozoon* group Microsporidia; GTR, generalized time reversible.

The short subunit (SSU) rRNA gene phylogeny in Fig 1 shows the relationship of *E*. *bieneusi* to its closest known relative, *Enterospora canceri*, a parasite of marine crabs, and all other characterised members of the EGM. Except for *E*. *bieneusi*, all members of the EGM are parasites of fish and aquatic invertebrates (including *Enterocytospora* and *Parahepatospora*, which do not branch robustly with the rest of EGM and so are not included in its circumscription here). The combined ecological and phylogenetic evidence suggests an aquatic origin of *E*. *bieneusi*, which unlike all other members of the clade (so far as is known), is the only EGM lineage to parasitise terrestrial warm-blooded vertebrates. However, it is unknown whether *E*. *bieneusi* has aquatic hosts. Notably, unlike other subbranches within microsporidian clade 4, the EGM has rarely been detected in environmental DNA (eDNA) studies [8], suggesting that their life cycles do not include small hosts or host-free stages that are usually sampled from water, sediments, and soils by such studies. Two exceptions to this are the *Daphnia*-derived lineages shown on Fig 1, but these also apparently have not yet been detected in eDNA studies. Other lineages such as *Desmozoon*/*Paranucleospora* are known to cycle between vertebrate and invertebrate hosts and could potentially be detected in eDNA studies [8]. It is possible that broadly targeted microsporidian-specific primers bias against detection of EGM; the use of clade-specific primers will mitigate against this. The question of when and how *E*. *bieneusi* effected the transition to terrestrial homeotherms can be addressed by further sampling of putative hosts and eDNA probing of relevant environments. It is important to distinguish between evolutionary and ecological transitions. In evolutionary terms, other currently unknown lineages may be discovered with a closer sister relationship to *E*. *bieneusi* than *E*. *canceri*. The host affiliation(s) and habitat of these lineages, if they exist, will be informative about evolutionary intermediates between the marine invertebrate–infecting *E*. *canceri* and *E*. *bieneusi*. An ecological approach would determine whether *E*. *bieneusi* is restricted to vertebrate hosts in nonmarine habitats, whether it infects marine mammals, and from a different perspective, whether *E*. *canceri* can also infect vertebrates or nonmarine animals. Parasite life cycles can also be investigated with eDNA methods, screening both potential hosts and environmental samples for insight into alternative hosts, vectors, and reservoirs. Freshwater and terrestrial habitats have been undersampled for Microsporidia by these approaches compared with marine systems.

## Deep obligates

Microsporidia are often used as examples of extreme metabolic streamlining in the eukaryotes. No other known eukaryotic group has a smaller number of protein-coding genes or a higher level of metabolic dependency on a host. This streamlining likely occurred in the ancestor of the Microsporidia after the divergence of the main group of microsporidia away from early-branching ‘intermediate’ Microsporidia such as *Nucleophaga*, *Paramicrosporidium*, and *Mitosporidium* [9]. Large numbers of genes were lost early in microsporidian evolution, followed by lineage-specific duplication of transporters and expansion of gene families according to the needs of the parasites and their relationships with their host [10]. Perhaps the most archetypal metabolic losses in the phylum are those of the tricarboxylic acid and electron transport chain and the ability to produce ATP in the mitochondria. This was potentially facilitated by the acquisition of ATP transporter by lateral gene transfer to uptake ATP and other nucleotides from the host [11]; however, in the spore stage, there is no access to host ATP, so here, glycolysis is the key pathway for intrinsic energy generation. The EGM has taken microsporidian reductionism a step further and no longer encodes the genes for glycolysis, making them utterly reliant on their hosts for energy [12]. Multiple genomes from the EGM have now been sequenced, and none of them encodes a functional glycolytic pathway, though curiously, each lineage retains genes for different parts of the pathway [13]. Nor do they have the genes for the pentose phosphate pathway or trehalose metabolism, and the capacity to metabolise fatty acids is severely reduced compared with other Microsporidia. What is different about EGM compared with other Microsporidia that may have allowed this metabolic degeneration? They are distinctive in their relationship to the host, either closely abutting the host nucleus or even exclusively living within it [14]. It might be intuitive to think that development in the nucleus eliminates access to the host mitochondria and thus a source of ATP (many Microsporidia gather host mitochondria around their cell); however, ATP levels are not thought to differ between nucleus and cytoplasm, suggesting neither advantage nor disadvantage to nuclear localisation with regard to energy acquisition [15]. However, several other microbes that are also energy parasites live within host nuclei [16]. Regardless of which selection pressures led to the loss of the glycolytic pathway in the EGM, it still leaves this group with the problem of not being able to generate/acquire energy in the spore stage, meaning that they do not have immediate access to ATP for the potentially energetic process of spore germination. To overcome this, future research will tell us whether they are able to either store ATP in the dormant spore, acquire ATP from the host as an endocytosed spore prior to germination, or encode a yet-unknown mechanism of generation of ATP from a storage product.

## Infection, disease, and host outcomes

Without exception, infection by members of the EGM is confined to the gastrointestinal tract and directly associated organs of their hosts. In humans, *E*. *bieneusi* infects the enterocytes of the superficial lining, rarely disseminating to fibrovascular tissues of the laminar propria [17], leading to self-limiting or persistent diarrhoea and wasting in immune-competent and immune-compromised patients, respectively [18]. Similarly, for fish, *Enterospora nucleophila* infects enterocytes and rodlet cells of the intestine, causing lethargy, stunting, and cachexia, resulting in emaciation [19]. In shrimp, *Enterocytozoon hepatopenaei* infects the epithelial cells of the hepatopancreas (a digestive organ associated with the gut), where it associates with a slow-growth syndrome [4], whereas in crabs, *E*. *canceri* infects the same cell type but resides almost exclusively within the nucleoplasm of host cells [14]. In all cases, systemic infection of other organ and tissue systems is not observed, suggesting that autoinfection (cell-to-cell within the gut) and transmission (via faeces) underpin the high prevalence observed in susceptible human and animal populations, as well as the role of food and water in spread [1]. Recent studies that challenge the concept that human enterocytes are nonphagocytic [20] coupled with those demonstrating potential for phagocytic uptake of some Microsporidia prior to their translocation across the cytoplasm in vacuoles [21] may indicate that EGM can be translocated by this mechanism into host cells. If so, this simple strategy, leading to infection of the primary contact layer within the host gut, could indicate almost sole reliance on the metabolic conditions therein to support their own spore germination and replication [13]. A key question relates to why exploitation of these apparently easy targets primarily occurs when these diverse hosts animals are immunocompromised.

## One health sentinels

The universal emergence of EGM is suggestive of multifactorial intestinal barrier dysfunction across susceptible host groups. Epithelial cells of the intestine form both metabolic and physical barriers to invasion by microbes and maintain an immunoregulatory function by influencing stasis of gut mucosal cells [22]. Direct disruption in this function can occur via diverse physical and psychological stressors [23] and, during ageing [24], lead to a multitude of disease outcomes in the host [25]. The infection of gut epithelial cells by EGM may then occur because of underlying immune, metabolic, or microbiological disruption (as proposed for co-incidence of *E*. *bieneusi* with immune-compromised human hosts [1]). In addition, infection with EGM may predispose hosts to direct and indirect effects of coinfection with other pathogens (e.g., infection with *E*. *hepatopenaei* makes shrimp more susceptible to effects of the bacterial agent of acute hepatopancreatic necrosis disease [AHPND] [26]). We have previously proposed that, for these reasons, opportunistic microsporidian parasites, epitomised by EGM, are living sentinels of host immune competence that traverse both host taxonomy and the biomes in which these hosts reside [1]. We predict that an increasing prevalence of immunosuppression in hosts from diverse systems—and driven by shifting demographic, environmental, pathophysiological, and psychological forces—will underpin further emergence of EGM in human and animal hosts. Understanding this emergence in the context of wider intestinal barrier dysfunction will be an inevitable focus of future research.
